# RNA Interference and CRISPR/Cas Gene Editing for Crop Improvement: Paradigm Shift towards Sustainable Agriculture

**DOI:** 10.3390/plants10091914

**Published:** 2021-09-14

**Authors:** Meenakshi Rajput, Khushboo Choudhary, Manish Kumar, V. Vivekanand, Aakash Chawade, Rodomiro Ortiz, Nidhi Pareek

**Affiliations:** 1 Department of Microbiology, School of Life Sciences, Central University of Rajasthan, Ajmer 305801, Rajasthan, India; 2019phdmb07@curaj.ac.in (M.R.); 2019phdmb0003@curaj.ac.in (K.C.); 2014phdmb04@curaj.ac.in (M.K.); 2 Centre for Energy and Environment, Malaviya National Institute of Technology, Jaipur 302017, Rajasthan, India; vivekanand.cee@mnit.ac.in; 3 Department of Plant Breeding, Swedish University of Agricultural Sciences, P.O. Box 101, 230 53 Alnarp, Sweden; rodomiro.ortiz@slu.se

**Keywords:** crop, CRISPR/Cas9, resistance, RNA interference, stress

## Abstract

With the rapid population growth, there is an urgent need for innovative crop improvement approaches to meet the increasing demand for food. Classical crop improvement approaches involve, however, a backbreaking process that cannot equipoise with increasing crop demand. RNA-based approaches i.e., RNAi-mediated gene regulation and the site-specific nuclease-based CRISPR/Cas9 system for gene editing has made advances in the efficient targeted modification in many crops for the higher yield and resistance to diseases and different stresses. In functional genomics, RNA interference (RNAi) is a propitious gene regulatory approach that plays a significant role in crop improvement by permitting the downregulation of gene expression by small molecules of interfering RNA without affecting the expression of other genes. Gene editing technologies viz. the clustered regularly interspaced short palindromic repeat (CRISPR)/CRISPR-associated protein (CRISPR/Cas) have appeared prominently as a powerful tool for precise targeted modification of nearly all crops’ genome sequences to generate variation and accelerate breeding efforts. In this regard, the review highlights the diverse roles and applications of RNAi and CRISPR/Cas9 system as powerful technologies to improve agronomically important plants to enhance crop yields and increase tolerance to environmental stress (biotic or abiotic). Ultimately, these technologies can prove to be important in view of global food security and sustainable agriculture.

## 1. Introduction

Food plays a vital role in the existence of human life on earth. With a rapidly growing population, it is, however, very difficult to fulfill the increasing demand for food globally by using traditional methods of crop improvement. People are making continuous efforts to improve crop yield, nutrient content, and to make disease-resistant crops by using conventional methods of crop improvement. Unfortunately, these plant breeding methods are not viable with the current needs of a fast-growing population as these approaches are laborious and time-consuming.

It has been evaluated that by the year 2050, there is an urgent need for increasing food production by 70% to feed the expanding population globally [1]. At present, a range of approaches such as crossbreeding, transgenic breeding and mutation breeding are in practice for the production of genotypes that are disease-resistant and resilient to climate change and other stresses. However, crossbreeding and mutation breeding are untargeted breeding methods with really backbreaking processes, while the production and commercialization process of the genotypes produced also faces many limitations, whereas in the case of transgenic breeding, apart from the long and costly commercialization process, genetically modified crops also encounter the challenge of public acceptance [2].

Recently, many advances have been made in the RNA-based gene regulation approach, i.e., RNA interference (RNAi), a gene regulatory tool that has been significantly diversified for crop improvement by modifying the expression of the gene for better trait quality with fewer biosafety issues as an expression of the transgene that is absent in transgenic lines. RNAi is a gene silencing phenomenon, which can be employed for the assessment of gene function, plant metabolic engineering, and in the development of stress-tolerant and disease-resistant crops [3].

Over the past five years, the RNA-guided nucleases-based gene editing approach i.e., the clustered regularly interspaced short palindromic repeat (CRISPR)/CRISPR-associated protein (CRISPR/Cas), has been recognized as an efficient tool for targeted gene editing in crops [4]. CRISPR allows targeting a sequence for gene knockin, knockout, and replacement along with observing and regulating gene expression by binding a specific sequence at the genome and epigenome levels. The genome editing function of CRISPR depends upon the three components viz. CRISPR RNA (crRNA), CRISPR-associated enzymes (Cas), and trans-activating crRNA (tracRNA). These three components can be constructed together to form a single chimeric synthetic RNA molecule known as single-guide RNA (sgRNA) for genome editing functions [4]. CRISPR provides the possibilities of targeting multiple genes simultaneously along with the ease of multiple editing. Thus, it has been widely used to edit, regulate, and monitor genes not only in plants but also in bacteria and animals. For genome modification, dsDNA breaks are introduced at specific locations by site-specific nucleases, which further stimulates DNA repair mechanisms, i.e., non-homologous end joining (NHEJ) and homology-directed repair (HDR) to introduce specific genome modifications. The NHEJ pathway works by ligating the broken ends of DSB without using homologous DNA, which results in insertions or deletions (InDels) or single-nucleotide polymorphism (SNP) at the cut site leading to frameshift or nonsense mutations. In the case of HDR, gene replacement takes place with the help of a homologous template at the breakpoint. Therefore, both NHEJ and HDR play an important role in nuclease-based gene editing [5]. In crop breeding, this approach generates the transgene-free bred cultivars. In this regard, this review encompasses various roles and possible applications of RNAi and the RNA-guided CRISPR/Cas9 system as powerful technologies to improve agronomically important crops to significantly enhance crop yields and tolerance to various environmental stress agents of both biotic and abiotic origin. Limitations, challenges, and potential future development have also been discussed.

## 2. RNA Interference

RNA interference is an evolutionarily conserved, naturally occurring, gene regulatory phenomenon in eukaryotic cells. It has been evolved to protect cells against invading foreign DNA. Besides this, it also helps in maintaining genomic stability, transposon movement regulation, epigenetic modification, and controls cellular processes at transcriptional and translational levels [6,7]. The gene silencing phenomenon was unfolded accidentally in *Petunia* flowers when Napoli et al. [8] were experimenting to deepen the color of petunia flowers by upregulating the gene coding for pigment production, which surprisingly resulted in variegated flowers instead of expected deep purple flowers. Since the expression of a homologous endogenous gene, as well as a transgene, was suppressed, the phenomenon was called “co-suppression” [8]. Fire et al. [9] discovered the same phenomenon in the nematode *Caenorhabditis elegans*, when they injected dsRNA in *C. elegans,* which resulted in efficient silencing of the target endogenous gene homologous to RNA, hence the phenomenon was named RNA interference (RNAi) [9]. This turned out to be one of the most compelling discoveries in biotechnology, because of its targeted gene regulation, accuracy, and heritability [10,11]. The gene expression in plants can be regulated through plant endogenous small RNAs (sRNAs) and it can be divided into endogenous short interfering RNAs (siRNAs) and microRNAs (miRNAs) [12]. The locus annotations of siRNAs are behind miRNAs, which have well-annotated loci. However, miRNAs consist of a small portion of the total sRNA pool. Moreover, miRNAs are more conserved as compared to siRNA across species [12]. The miRNAs can be applied to achieve simultaneous silencing of multiple targets through the production of polycistronic miRNA precursors [13]. Moreover, the segregation of the RNAi transgene has been reported to produce non-genetic MSH1 (a plant-specific mitrochondrial-and plastid-targeting protein) memory, which can be inherited in multiple generations [14]. The study suggested that RNAi suppression of MSH1 could lead to inconsistency in the phenotype related to the developmental and stress response pathways.

Similar mechanisms have also been observed in fungi as “quelling” [15] bacteria such as the CRISPR/Cas system [16], algae [17], fruit fly [18], and mammals [19]. Since then, research in this field has been burgeoning and researchers feel that RNAi is a promising tool for gene regulation with greater potential as compared to other post-transcriptional gene regulation technologies such as antisense technology. RNAi is a naturally occurring phenomenon in eukaryotes with its oldest and omnipresent antiviral defense system, whereas almost all antisense RNAs are found in prokaryotes [20].

In this biological process, small non-coding RNAs (21–28 nt. long), which participate in the gene regulation, are the cleavage product of dsRNAs, i.e., microRNA (miRNA) and small interfering RNA (Si RNA). The process of cleavage is carried out by a multidomain endoribonuclease named Dicer or the Dicer-like enzyme, which belongs to the RNase III family [21]. Finally, these small non-coding RNAs (ncRNA) are associated with the RNA-induced silencing complex (RISC), argonaute (AGO) [22], and other effector proteins, and cause complex degradation of the target messenger RNA [16,23]. Thus, RNAi can be defined as the capability of endogenous or exogenous dsRNA to inhibit the expression of the gene whose sequence is complementary to dsRNA [24].

### 2.1. RNAi Mechanism

#### 2.1.1. Components of RNAi Machinery

Two ribonucleases participate in the RNAi pathway—first, Dicer and second, the RNA-induced silencing complex (RISC), where Dicer cleaves the dsRNA into active small non-coding RNAs and initiates the RNAi pathway [21], while RISC with the RNase H core enzyme Argonaute (AGO) accomplishes the gene silencing [22]. The Dicer family belongs to the class 3 RNase III enzyme and consists of four domains: N-terminal helicase domain, a PAZ (Piwi/Argonaute/Zwille) domain, dual RNase III domains, and a dsRNA binding domain. The primary function of these enzymes is to recognize the dsRNA precursor from the RNAi pathway and to generate small non-coding RNA of a specific length (21–24 nt long). The Dicer catalysis model proposes that in the multidomain dicer enzyme, two RNase III domains dimerize and form an intramolecular pseudo-dimer, which serves as the active center. It has also been suggested that each domain cuts a single strand of dsRNA, forming a new terminus [25]. Finally, the last step of the RNAi pathway, i.e., gene silencing by target mRNA degradation, is performed by RISC in association with the argonaute (AGO) protein and other effector proteins. Argonaute proteins are primarily found in bacteria, archaea, and eukaryotes. The significant function of the Argonaute protein is to recognize guide strand termini, cleave the target mRNA with its nuclease activity, or recruit other proteins involved in silencing. RISC with gene silencing also participates in the cellular surveillance process [16,20].

#### 2.1.2. Mechanism of Action

Over the last two decades, the functionality of small non-coding RNA in gene regulatory processes of transcriptional gene silencing (TGS) and post-transcriptional gene silencing (PTGS) has continuously been explored. Various classes of small non-coding RNAs have been discovered so far. These include miRNA, siRNA, piRNA (PIWI–interacting RNA), qiRNA (QDE-2-interacting RNA), svRNA (small vault RNA), etc., having different biogenesis pathways and regulatory mechanisms [26]. Initially, the process of biogenesis of miRNA and siRNA differs to form their corresponding dsRNA precursors as the cellular origin of miRNA is the genomic DNA, whereas siRNA can be generated endogenously via cleavage of dsRNA into smaller segments or it can be exogenously derived directly from the viruses, transposons, or transgene. Regardless of these differences, they have similarities in their sizes and sequence-specific inhibitory functions, which clearly suggest that their respective biogenesis pathways and mechanisms are related to each other somehow. The RNAi pathway comprised four steps: The formation of snRNA as a cleavage product of dicer, loading of snRNA into the RISC complex, activation of the silencing complex, and target mRNA degradation [20].

### 2.2. Micro RNA (miRNA)

**Figure 1:**
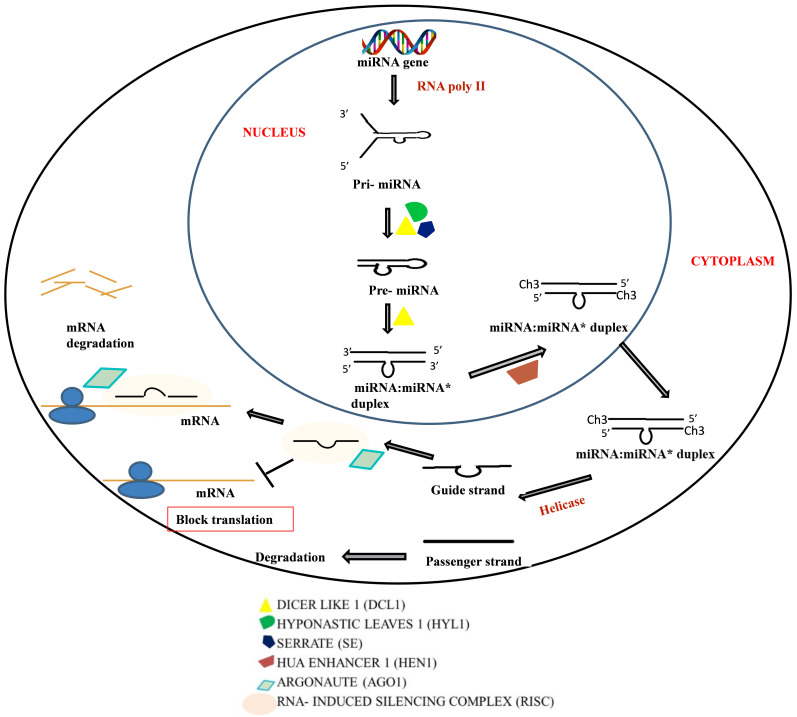
Mechanism of miRNA biogenesis and gene silencing. The miRNA biogenesis commences with the transcription of miRNA genes into pri-miRNA by RNA polymerase II, which is further subjected to primary and secondary processing via the enzyme complex of DCL-1, SE, and HYL1 leading to the generation of pre-miRNA and an miRNA/miRNA* duplex, respectively. Further the HEN1-mediated methylation at the 3′-OH end leads to the export of the duplex to the cytoplasm. In the cytoplasm, the passage RNA strand is degraded and the guide strand from the miRNA-inducing silencing complex (miRISC) directs the degradation or translation inhibition of the target mRNA.

miRNAs are 21–24 nucleotide (nt)-long small RNAs, which are derived from MIR genes. The biogenesis of miRNA occurs in the nucleus by RNA polymerase II aided transcription of MIR genes, forming a primary miRNA (pri-miRNA) transcript of about 1000 nt (Figure 1). Due to the presence of intramolecular sequence complementarity in pri-miRNA, an imperfect folded-back stem-loop or hairpin structure formation takes place, which is further processed into a short stem-loop precursor known as pre-miRNA with the aid of DCL1 assisted by the dsRNA binding protein DRB1or HYL1 [27]. This pre-miRNA is again cropped by DCL1 in the nucleus and generates the RNA duplex (miRNA:miRNA*), which consists of mature miRNA (guide strand) and miRNA* (passenger strand) [28]. The 3′-terminals of the RNA duplex get methylated by HUA ENHANCER (HEN1) at the 2′-O- hydroxyl group to prevent degradation of miRNA:miRNA* [29,30]. After methylation, the RNA duplex is exported to the cytoplasm where mature miRNA is loaded onto the RISC complex with AGO and other effector proteins. This miRNA-induced silencing complex (miRISC) base pairs with the complementary target mRNA completely, then the AGO protein with its characteristic nuclease activity degrades the target mRNA [31]. In the case that complete base pairing does not occur between miRISC and the target mRNA, then miRISC inhibits the translation process.

In 2011, Huntzinger and Izaurralde suggested that miRNA-mediated downregulation of gene expression occurs by (1) miRISC-mediated inhibition of translational initiation or ribosome subunit joining, premature degradation of the budding polypeptide chain, and an increase in drop off of the ribosome; or (2) inducing deadenylation and destabilization of the target mRNA [32]. Expression of miRNA is usually witnessed during the phase of plant growth and development, secondary metabolite synthesis, abiotic and biotic stress, etc. Hence, a change in expression and biogenesis of these RNAs could lead to the formation of the crop with agronomically valuable characteristics [33].

### 2.3. Small Interfering RNA (siRNA)

**Figure 2:**
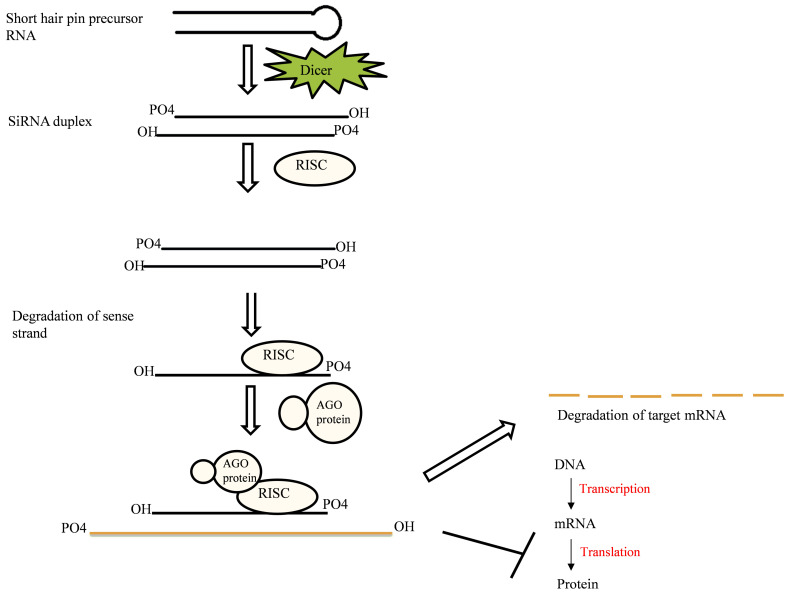
siRNA biogenesis and gene silencing. Here the action of Dicer or the Dicer-like enzyme on the precursor RNA results in an siRNA duplex with overhangs at 3′-OH ends. Further, the antisense strand from siRNA induces the silencing complex by associating with the RISC protein. Thereafter, incorporation of AGO and other effector proteins with siRISC facilitate the gene silencing through degradation of the target mRNA or translation inhibition.

Gene silencing through RNAi can be triggered via long dsRNA or short hairpin precursors, which can perfectly base pairs with the gene to be silenced. The introduction of long endogenous dsRNA directly into the cytoplasm or access of transgene, viral intruders, or transposable elements can ignite the RNAi pathway by recruiting the Dicer or Dicer-like enzymes [34]. This Dicer enzyme crops these dsRNAs into short 21–24 nt long SiRNA duplexes with 2nt overhangs at the 3′OH end and 5′ phosphorylated ends [35,36]. Thereafter, the SiRNA-induced silencing complex (SiRISC) is recruited and degrades the sense strand (has precisely the same sequence as that of target mRNA) of SiRNA, whereas the antisense strand of siRNA along with siRISC get loaded onto the target mRNA in a sequence-specific manner (Figure 2). siRISC incorporation with the AGO protein and other effector proteins leads to post-transcriptional gene silencing (PTGS) by cleavage of the target mRNA or inhibition of translation [37]. Aside from this, siRNAs by chromatin regulation can also participate in the co-transcriptional gene silencing. Dicer-independent siRNA genesis has also been reported in *Neurospora*, *C. elegans*, *Schizosaccharomyces pombe*, *and Arabidopsis* [38,39,40,41]. These dicer-independent siRNAs mostly arise from transposable elements, intergenic elements, and transgenes [41].

### 2.4. Role of RNAi in Crop Improvement

In the 21st century, one of the major goals is to provide food security and stop the malnutrition across the world, but factors such as abiotic and biotic stresses, anthropogenic effects, climate change, and depletion of natural resources limit the crop production globally [1,42]. Thus, to overcome these problems, genetic engineering should be used in a way to manipulate the physiology of plants, genomes, and proteomes. In this context, RNAi has been extensively explored by researchers for improving a range of crop features including stress tolerance, disease resistance, yield enhancement, etc. (Table 1).

#### 2.4.1. Biotic Stress Resistance

In plants, biotic stress is caused by living organisms, especially viruses, bacteria, fungi, insects, arachnids, nematodes, and weeds. These organisms account for about a 40% loss in the overall yield of six major food and cash crops [91]. RNAi technology has opened up new prospects for crop protection against biotic stresses.

##### RNAi-Mediated Virus Resistance

Viruses are the leading agents behind the major loss of crop productivity as it is very difficult to control them due to their diverse strategies to multiply and transmit diseases in the host plant. Therefore, pathogen-derived resistance (PDR) has been considered as one of the most efficient approaches in fighting viral infections in plants. However, there is one more approach, i.e., RNA interference that provides broad-spectrum resistance against viral infections by targeting multiple regions of a viral gene. It relies on the principle of targeted silencing of the viral coat proteins (CP). In 1998, the first RNAi-mediated virus-resistant potato transgenic lines were reported, which were transformed by simultaneous expression of both sense and antisense transcripts of the helper-component (HC-Pr) gene and showed complete resistance against *Potato virus Y* (PVY) [92]. Missiou et al. [93] developed potato transgenic lines that were highly resistant to three strains of PVY by expressing the dsRNA derived from the 3′-terminal end part of viral coat proteins (CP), which has been reported as the highly conserved region of PVY isolates. Over recent years, many RNAi-mediated virus-resistant cultivars targeting the viral coat protein have been produced i.e., *Beet necrotic yellow vein virus* (BNYVV)-resistant tobacco [94], *Plum pox virus* (PPV)-resistant *Prunus domestica* and *N. benthamiana* [95], and *Bean golden mosaic virus* (BGMV)-resistant *Phaseolus vulgaris* [96].

Si-RNA-mediated silencing of the *African cassava mosaic virus* (ACMV) by targeting the replication-associated protein 1 (AC1) resulted in a 66% decrease in ACMV genomic DNA [97]. In 2009, Vanderschuren et al. [98] performed an experiment in which they developed dose-dependent RNAi-mediated transgenic cassava lines resistant to ACMV. Cassava brown streak disease (CBSD) is considered one of the threats for cassava (*Manihot esculenta)* cultivation in East Africa. In this regard, Patil et al. [99] first developed cassava plants resistant to CBSD and provided protection against two causative organisms belonging to two different species, i.e., the *Cassava brown streak* virus (CBSV) and the *Cassava brown streak Uganda virus* (CBSUV) by using RNAi construct containing 397-nt from N-terminal end and 491-nt from the C-terminal end of the coat protein gene of the viruses [99].

*Tobacco streak virus* (TSV)-resistant transgenic lines of both tobacco and sunflower (*Helianthus annuus* L.) were produced by RNAi technology using a 421-bp-long coat protein gene containing both sense and anti-sense coat protein sequences [100]. Rice strip disease caused by the *Rice strip virus* (RSV) was successfully suppressed in two RSV-susceptible varieties of *Japonica* by RNAi construct consisting of the CP gene and the disease-specific (SP) gene [101]. *Soybean mosaic virus* (SMV)-resistant transgenic lines of soybean were produced by introducing a hairpin RNAi construct containing the Hc-Pro gene [102]. Peanut (*Arachis hypogaea* L.) plants resistant to the *Tobacco streak virus* were developed using hairpin RNA comprising TSV-coat proteins.

Pooggin et al. [103] demonstrated that DNA of a replicating virus can be used as an RNAi target. They used this approach in the silencing gene associated with bidirectional promoters and witnessed recovery from infection of the *Mungbean yellow mosaic India virus* (MYMIV) in *Vigna mungo*.

A study conducted on *N. benthamiana* and *Vigna unguiculata* plants to develop resistance against the *Bean common mosaic virus* (BCMV) by exogenous application of RNAi construct containing viral coat proteins to protect plants from aphid mediated transmission of BCMV [104]. *Rice tungroo bacilliform virus* (RTBV)- and *Rice tungroo spherical virus* (RTSV)-resistant *O. sativa* cultivars have been developed by using a highly conserved *coat protein 3* (*CP3*) gene in an RNAi construct. They observed high resistance in *O. sativa* against tungroo disease, and the ability to transmit the virus has also been decreased in transgenic lines [50].

##### RNAi-Mediated Bacterial Resistance

Bacteria serve as the biggest hurdle in crop production as they are ubiquitous in nature as well as replicating with great speed and causing infection. Hence, it is important to produce bacterial-resistant crops. Escobar et al. [53] conducted a study on *A. thaliana* and *S. lycopersicum* (tomato) to suppress crown gall disease caused by *Agarobacterium tuminifaciens* through RNAi technology. For this, they designed dsRNA construct homologous to oncogenes iaaM and ipt, which are necessary for tumor formation. Katiyar-Agarwal et al. [54] demonstrated that *P. syringae* infection in *A. thaliana* induced biogenesis of endogenous si-RNA i.e., nat-SiRNAATGB2. This siRNA downregulated the expression of the PPRL gene, which is considered a negative regulator of the RPS2 resistance pathway.

##### RNAi-Mediated Fungal Resistance

Research findings suggest that RNAi technology can be used to enhance resistance against fungi in genetically engineered crops. Gene silencing has been studied using homologous transgenes (co-suppression), antisense or dsRNAs in many plant-pathogenic fungi such as *Cladosporium fulvum* [105], *Venturia inaequalis* [106], *N. crassa* [107], *and Magnaporthe oryzae* [108]. Enhancement of resistance against *Phytophthora parasitica var. nicotianae* has been observed in *N. tobacum* by downregulation of the GST (glutathione S-transferase) enzyme gene via RNAi [109]. Fusarium wilt has been classified among the most destructive diseases of banana, caused by *F. oxysporum* f. sp. *Cubense* (Foc). Banana transgenic lines developed by intron hairpin RNA (ihp-RNA)-mediated expression of si-RNA has shown enhancement in resistance against Foc. This was achieved by the downregulation of a vital gene of Foc fungus and confirmed by performing 6-week-long bioassays in the greenhouse [58]. Chen et al. [110] explored the role of RNAi machinery in the causative agent of wheat head blight, i.e., *Fusarium graminearum* by the aid of hpRNA for silencing the target mRNA. They also studied the importance of FgAgo (Argonaute protein) and FgDicer2 in gene silencing.

*Agarobacterium*-mediated transformation (AMT) of RNAi constructs act as a potent approach for investigating the role of the gene involved in pathogenesis. Transformation of *F. oxysporum* spores using RNAi construct of three MAP Kinase signaling genes (viz. *Fmk1*, *Hof1,* and *Pbs2*) via AMT resulted in reduced invasive growth on tomato fruits, pathogenesis, loss of surface hydrophobicity, and hypo-virulence on tomato seedlings [64].

Functional analysis of the membrane-localized gene GmSnRK1.1, important for soybean resistance against *Phytophthora sojae,* has been done by overexpressing the gene and RNAi silencing. Results obtained show that overexpression of genes increase the resistance whereas RNAi-mediated silencing leads to a reduction in resistance [67].

##### RNAi-Mediated Insects and Nematode Resistance

Insects and nematodes are capable of causing severe damage to the crops. Some of the most disastrous nematodes are *Meloidogyne* spp., *Heterodera* and *Globodera* spp., *Pratylenchus* spp., *Helicotylenchus* spp., *Radopholus similis*, *Ditylenchus dipsaci*, *Rotylenchulus reniformis*, *Xiphinema* spp., and *Aphelenchoides* spp. [111]. Gheysen and Vanholme [112] suggested that the expression of dsRNA targeting the housekeeping gene and the parasitism gene of root-knot nematodes (RKN) in a host plant led to resistance against nematode infection. Bioengineering of crops expressing dsRNA that targets the RKN parasitism gene could help in providing broad-spectrum resistance to crop against RKN [113].

Cyst nematodes are considered to be highly evolved sedentary endoparasites that cause great damage to the crops globally. Through host-induced RNAi silencing, all four parasitism genes of the sugar beet cyst nematode (*Heterodera schachtii*) were targeted with *A. thaliana* as the host, resulting in a decrease in the number of female nematodes. No complete resistance was observed, however, but the reduction in developing nematodes ranges 23–64% in different RNAi lines [114]. Similarly, enhanced resistance against the soybean cyst nematode *H. glycines* has been reported by targeting reproduction and fitness-related genes, i.e., *HgY25* and *HgPrp17* in soybean transgenic lines [78].

RNAi-mediated root-knot nematode (*Meloidogyne incognita)* resistance was pursued in *A. thaliana* for targeting two housekeeping genes i.e., splicing factor (349 bp) and integrase enzymes (624 bp). Splicing factor and integrase enzyme are important for nematodes as they play a prominent role in RNA metabolism. Hence, their RNAi-mediated silencing resulted in a significant decrease in the number of galls, females, and egg masses [115]. Tsygankova et al. [116] examined RNAi-mediated invitro resistance in bread wheat (*T. aestivum* L.) against the parasitic nematode *H. avenae* using bioregulators of microbial origin.

The success of the cry toxin from *Bacillus thuringiensis* as an insecticide has led to the foundation of RNAi-mediated insect resistance in crops. The RNAi technology came into the limelight when two reports regarding insect control occurred in the scientific community. Mao et al. [117] developed transgenic lines of *Arabidopsis* and tobacco plants expressing CYP6AE14- specific dsRNA. In cotton worm, this gene confers resistance against gossypol, a polyphenol compound. When cotton bollworm larvae fed on leaves of transgenic lines, they showed sensitivity against gossypol in an artificial diet. Baum et al. [118] developed transgenic maize lines resistant to western corn rootworm by expressing dsRNA specific to the gene encoding the A subunit of V-type ATPase pump. Thus, these results suggested that the RNAi pathway can be exploited to control pests from harming the plants by targeting the significant gene of insects. V-type ATPase subunit-A coding genes were also used crucial to develop resistance against the whitefly *(B. tabaci)* population in tobacco plants [119]. Likewise, RNAi-mediated whitefly (*B. tabaci*)-resistant transgenic lines of lettuce (*Lactuca sativa*) targeting V-ATPase transcripts in the whitefly increased the mortality rate of insects feeding on transgenic plants from 83.8−98.1% [71].

Wang et al. [120] reported that 3-hydroxy-3-methyl glutaryl coenzymeA reductase (HMG-CoA reductase, HMGR) is a significant enzyme in the insect mevalonate pathway and can be utilized as a potential target to produce insect-resistant cultivars using RNAi. Similarly, the chitinase (HaCHI) gene important for molting in insects can also be used as a target to produce insect-resistant plants. Through host-induced RNAi, Helicoverpa armigera-resistant transgenic tobacco and tomato plants were developed [69].

#### 2.4.2. Abiotic Stress Tolerance

Plants in their natural field conditions constantly get exposed to various abiotic factors such as high salinity, variation in temperature, flood, drought, and heavy metals, which hinders proper growth and development in plants. These factors are also one of the major causes behind huge crop losses globally. The changing climatic conditions and rapidly expanding population demand creates an urgent need to develop more stress-tolerant cultivars. Hence, RNA interference technology can be exploited to develop transgenic cultivars that can cope with different abiotic stresses. Functional genomics studies revealed that novel genetic determinants are involved in stress adaptation in plants, which can be employed to attain stress tolerance [121].

The receptor for activated C-kinase 1(RACK-1) is a highly conserved scaffold protein that plays a significant role in plant growth and development. RNAi-mediated downregulation of *RACK-1* gene in transgenic *O. sativa* plants has shown more tolerance to drought stress as compared to the non-transgenic *O. sativa* plants [122]. Likewise, disruption of *O. sativa* farnesyltransferase/squalene synthase (SQS) by maize squalene synthase via RNAi resulted in enhanced drought tolerance at the vegetative and reproductive stages [123].

Stress tolerance and development in plants are regulated by miRNA, also negatively affecting the expression of the post-transcriptional gene. Wang et al. [124] examined that miRNAs are involved in the very early stage during seed germination and identified that miRNA-mediated regulation of gene expression is present in maize imbibed seed. Wang et al. [125] reported 32 known members of 10 miRNA families and 8 new miRNAs/new members of known miRNA families that were found to be responsive to drought stress by high-throughput sequencing of small RNAs from *Medicago truncatula*. These findings suggest the importance of miRNAs in the response of plants to abiotic stress in general and drought stress in particular.

*OsTZF1* is a member of the CCCH-type zinc finger gene family in rice (*O. sativa*). Conditions like drought, high-salt stress, and hydrogen peroxide can induce the expression of *OsTZF1*. Expression of *OsTZF1* gene was also induced by abscisic acid, methyl jasmonate, and salicylic acid. The *OsTZF1* gene overexpressed in transgenic plants showed enhanced tolerance to high salt and drought stresses, whereas transgenic *O. sativa* plants in *OsTZF1* gene silenced using RNAi technology has shown less tolerance. This suggests the role played by *OsTZF1* gene in abiotic stress tolerance [126].

Dehydrin proteins play a significant role in protecting plants from osmotic damage. Various research results suggest that the overexpression of the dehydrin gene *WZY2* provides more tolerance for plants against osmotic stress. A study conducted by Yu et al. [127] suggests that RNAi-mediated silencing of the *WZY2* gene in *A. thaliana* makes plants intolerant to osmotic stress.

#### 2.4.3. Seedless Fruit Development

Seedless fruits are generally appreciated by the consumers as seedlessness increases the quality of fruit with the enhancement of shelf life [128,129]. Seedless fruits can be obtained by parthenocarpy, which involves the development of fruit directly from the ovary without fertilization. In eggplant, seedlessness prevents browning and texture reduction of pulp [130]. The production of seedless fruits can be induced artificially by disrupting the genes involved in the formation process of seed and seed set. The seed formation process is regulated by the phytohormones both temporally and spatially. Generally, seedless fruit obtained by inducing mutation or alteration in phytohormones shows pleiotropic effects i.e., change in taste, reduced fruit size, etc. Hence, for the production of parthenocarpic fruits, novel methods with more efficiency should be employed [130]. It has been shown that seed development in fruits limits the yield in cucumber [131,132] and tomato [133]. Thus, the replacement of seed and seed cavities with edible fruit tissue is highly desirable and appreciated by consumers, breeding companies, and production companies. Auxin response factors (ARFs) encode transcription factors that control auxin-dependent plant developmental processes. ARF7 factor of tomato (*S. lycopersicum*) designated as SlARF7 was found to be highly expressed in unpollinated mature ovaries. Further research revealed that the expression of slARF7 remains high from the initial period of flower development to the formation of mature flowers and decreases within 48 h after pollination. RNAi-mediated development of transgenic tomato lines with a downregulated *slARF7* gene resulted in the generation of parthenocarpic fruits [134]. Schijlen et al. [135] developed seedless tomatoes through RNAi-mediated suppression of the chalcone synthase (CHS) gene, the first gene used in the flavonoid synthesis pathway. Likewise, post-transcriptional gene silencing of the *flavonol synthase* (*FLS*) gene, a vital enzyme for flavonols production, resulted in the generation of seedless or less-seeded fruits in tobacco (*N. tobacum* cv *xanthi*) [136].

Aucsia genes are distinctly expressed in auxin biosynthesis parthenocarpic flower buds of tomato. The silencing of these genes by RNA interference resulted in parthenocarpic fruit development in tomato with some other auxin-related phenotypes [137]. Takei et al. [138] isolated and characterized small parthenocarpic fruit and flower (spff) mutants in a tomato cultivar. Linkage analysis and RNAi-based silencing of the *Solyco4g077010* gene, which encodes the receptor-like protein kinase, resulted in impaired male sterility with parthenocarpic fruit set development.

#### 2.4.4. Shelf-Life Enhancement

Fruits and vegetables are more vulnerable to spoilage as compared to cereals because of their nature and composition. Despite being one of the leading producers of fruits and vegetables, India faces massive losses due to post-harvest mishandling, spoilage, and pest invasion during storage and transportation. Hence, it is essential to augment the shelf-life of fruits and vegetables to minimize horticultural losses. This can be achieved by delaying the ripening of the fruit by regulating ethylene biosynthesis, ethylene-mediated signaling, and ethylene response elements with the aid of RNAi. In contrast to other phytohormones, ethylene is the gaseous hormone that plays a major role in the process of fruit ripening through a cascade of signals. 1-Aminocyclopropane-1-carboxylate (ACC) oxidase is an enzyme that catalyzes the biosynthesis of ethylene from its precursor ACC. Tomato transgenic lines with enhanced shelf life have been developed by RNAi-facilitated suppression of ACC oxidase enzyme [139]. Similarly, the expression of three homologs of 1-Aminocyclopropane-1-carboxylate synthase (ACS) was suppressed during the period of ripening in tomato fruits, thereby leading to the production of delayed ripening tomato fruits due to the inhibition of ethylene production [140]. Meli et al. [141] have identified and targeted two ripening-specific N-glycoprotein modified genes, α-mannosidase (α-Man) and β-D-N-acetylhexoaminidase (β-Hex), and their suppression via RNAi resulted in fruit softening with an extended shelf life.

The *SISGR1* gene encodes for the STAY GREEN protein, which regulates fruit color development and ripening by altering ethylene signal transduction in tomatoes. Fruit shelf-life was found to be extended in *SISGR1*-gene-suppressed transgenic tomato lines [142]. Repression of two banana E class (*SEPALLATA3*) MADS box genes, i.e., *MaMADS1* and *MaMADS2,* through RNAi resulted in transgenic bananas (*Musa ascuminata*) having desirable characteristics such as delayed color development, reduced fruit softening, delayed ripening and extended shelf-life [143]. Yang et al. [144] reported 22 individual pectate lysase genes in tomato, out of which one pectate lysase gene, i.e., *SIPL*, has been found to be dominantly expressed during fruit maturation. RNA interference studies of *SIPL* revealed that it plays a significant role in the enhancement of fruit firmness, pathogen resistance, and prolongation of shelf life. Similarly, carotenoid and flavonoid content in tomatoes was increased by knocking down the endogenous photomorphogenesis regulatory gene *DET1* using RNAi [145]. The phenomenon of accumulation of sucrose and other reducing sugars in potato tubers during storage at low temperatures is called ‘cold sweetening’. In potato tubers, sugar phosphatase (SPP) plays a significant role in carbohydrate metabolism at room temperature. Downregulation of the SPP gene through RNAi leads to inhibition of cold-induced hexogenesis in transgenic tubers [146].

#### 2.4.5. Male Sterile Plants Development

The development of hybrid cultivars has augmented productivity due to hybrid vigor and improved uniformity. Hybrid production depends on the development of male sterility in one parent to ensure purity in hybrids for further production of hybrid seeds. Several methods involving conventional as well as genetic engineering has been reported for the production of male-sterile plants; however, RNAi has turned out to be one of the most efficient tools in the development of male sterile lines by targeting male-specific genes that participate in tapetum and pollen development. In tobacco plants, TA29, a male-specific gene expressed in anthers during microspore development has been targeted using RNAi technology, giving rise to transgenic male sterile lines [147]. Likewise, the downregulation of the Bcp1 gene of *A. thaliana* expressed in both diploid tapetum and haploid microspore resulted in the generation of transgenic male-sterile plants [148]. S-adenosylmethionine decarboxylase (SAMDC) is considered a significant enzyme in the biosynthesis of polyamines in tomato plants. Suppression of *SAMDC* gene in the tapetal tissue of tomato plants leads to the development of male sterility [149].

Cytoplasmic male sterility is the maternally inherited phenomenon present in plants. Nuclear genes play a crucial role in the rearrangement of mitochondrial DNA, which is found to be associated with the naturally occurring phenomenon of cytoplasmic male sterility during plant development. Suppression of *Msh-1*—a nuclear gene in tobacco and tomato plants—resulted in reproducible mitochondrial DNA rearrangement with male-sterility [150].

#### 2.4.6. Flower Color Modification

Floriculture or flower farming is a field of horticulture that deals with flowers and ornamental plant cultivation. Nowadays, the demand for flowers in different colors and patterns has increased for the purpose of decoration and scents. This can be achieved by silencing the pigment encoding genes using RNA interference technology. A cDNA encoding the *chalcone isomerase* (*CHI*) gene isolated from petals of *N. tobacum* was suppressed using RNAi, thus reducing pigmentation and altering flavonoid components in flower petals [151]. Similarly, flower color alteration in liliaceous ornamental *Tricyrtis* sp. has been reported using the RNAi construct TrCHS1 targeting *chalcone synthase* (*CHS*) [152]. RNAi-facilitated suppression of three anthocyanin biosynthetic genes, *chalcone synthase* (*CHS*), *anthocyanidin synthase* (*ANS*), and *flavonoid 3′*,*5′-hydroxylase* (*F3′5′H*), led to changes in flower color of ornamental gentian plants [153]. Naturally, the flower of gentian plants is vivid blue in color. The accumulation of a polyacrylate delphinidin ‘gentiodelphin’ in the petals of gentian plants contribute to the flower color. Anthocyanin 5,3′-aromatic acyltransferase (5/3′AT) and flavonoid 3′,5′-hydroxylase (F3′5′H) are crucial enzymes for gentiodelphin biosynthesis and their downregulation via RNAi causes modification in flower color [154].

He et al. [155] experimented to rebuild the delphinidin pathway, for which they first identified two cultivars of chrysanthemum and isolated seven anthocyanin biosynthesis genes, namely *CmCHS*, *CmF3H*, *CmF3′H*, CmDFR, *CmANS*, *CmCHI*, and *Cm3GT*. Furthermore, the overexpression of the *Senecio cruentus F3*′*5*′*H* (*PCFH*) gene and suppression of the *CmF3′H* gene in chrysanthemum resulted in increased cyanidin content with brighter red flower petals, but the accumulation of delphinidin has not been reported.

#### 2.4.7. Nutritional Improvement

Plants serve as the major source of required nutrients in the human diet. However, more than two-thirds of the world’s population is deficient in one or more essential mineral elements [156]. RNAi can be employed to achieve the required levels of nutrients in crops by modifying various biochemical and physiological pathways. Omega-3 fatty acid desaturase (FAD3) enzyme catalyzes the synthesis of α-linolenic acid (18:3) in the polyunsaturated fatty acid synthesis pathway. α-linolenic acid is responsible for instability in soybean (*Glycine* max) and other seed oils. Flores et al. [157], through siRNA-mediated silencing of FAD3 in soybeans, significantly decreased the level of α-linolenic acid by 1–3% as compared to other non-transgenic lines. In *Camelina sativa*, the oilseed quality has been improved by downregulating the *fatty acyl-ACPthioesterase* (*FATB*) gene using artificial miRNA (amiFATB). The results showed a considerable decrease in total saturated fatty acids content with a 45% reduction in palmitic acid (16:0) and a 38% reduction in stearic acid (18:0) as compared to wild-type seeds [158].

Kusaba et al. [159] generated an *O. sativa* cultivar with low glutenin content (named as LGC-1) through the silencing of the *gluB* gene using hairpin RNA. A high amylose content *Triticum* cultivar has been produced by suppressing the expression of two starch branching enzyme (SBE) II (namely SBEIIa and SBEIIb) in *Triticum* endosperm using RNAi [160]. In plants, starch phosphorylation and starch dephosphorylation act as crucial components in the starch degradation process. Downregulation of *glucan water dikinase* (*GWD*) and *phosphoglucan phosphatase* (*SEX4*) through RNAi resulted in the accumulation of starch in leaves of *A. thaliana* and *Z. mays* [161]. Carotenoid content in *Brassica napus* was elevated by RNA-mediated silencing *of* *ε-Cyclases* (*ε*-CYC). Seeds obtained by RNAi transgenic *Brassica* lines were found to be rich in β-carotene, zeaxanthin, lutein, and violaxanthin [162].

RNAi can also be employed for the accumulation of minerals in crops. Aggarwal et al. [163], through RNAi-mediated downregulation of *inositol pentakisphosphate kinase* (*IPK1*), produced *Triticum* grains with high Zn and Fe content with a reduced level of antinutrient phytic acid (PA).

#### 2.4.8. Secondary Metabolite Production

Plant secondary metabolites are used in fragrances, drugs, food additives, pigments, and pesticides. Biosynthesis of secondary metabolites is regulated by an array of multiple genes, but sometimes it may get obstructed by certain undesirable compounds. RNAi can be used as an effective approach to suppress the expression of those compounds as well as for secondary metabolite manipulation [164]. Allen et al. [165] reported the replacement of morphine with non-narcotic alkaloid (S)-reticuline in the opium poppy (*Papaver somniferum*) through RNAi-mediated silencing of multiple genes participating at different steps in a complex biochemical pathway. They constructed hpRNA for suppressing the expression of all the members of the codeine reductase (COR) gene family. This led to the development of transgenic lines consisting of (S)-reticuline, a non-narcotic alkaloid precursor, by replacing morphine, codeine, and opium.

Caffeine acts as a natural stimulant for the central nervous system, respiratory system, and circulatory system. It also lessens the risk of liver cancer, mouth, and throat cancer. Besides this, its excess intake may cause some health issues such as insomnia, nervousness, an upset stomach, restlessness, and muscle tremors. In coffee plants, three enzymes participate in the caffeine biosynthesis viz. CaXMT1, CaMXMT1 (theobromine synthase), and CaDXMT1 (caffeine synthase). The RNAi-mediated silencing of the *CaMXMT1* gene resulted in a 70% decrease in caffeine content, indicating RNAi technology can be employed for the production of decaffeinated coffee beans [166]. Similarly, low-caffeine-content-containing tea (*Camellia sinensis*) transgenic lines were developed by downregulating the caffeine synthase (CS) gene using RNAi [167].

*Salvia miltiorrhiza* is a famous Chinese herb, also used in other Asian countries. The production of phenolic acid was enhanced by downregulating the initial enzyme in flavonoid biosynthesis, i.e., the *Chalcone synthase* (*CHS*) gene with elicitor treatment of salicylic acid. Results showed a considerable decrease in flavonoid production with an increase in phenolic acid content [168]. In several aromatic plants such as spearmint (*Mentha spicata*), tiny, specialized structures are present for secondary metabolite production called peltate glandular trichomes (PGT). Wang et al. [169] examined the role of transcription factors in the secondary metabolite biosynthesis pathway, for which they isolated and functionally characterized a *MsYABBY5* gene expressed in PGT. The production of terpenes was increased after the suppression of the *MsYABBY5* gene, suggesting that encoded transcription factors act as a negative regulator for secondary metabolite production.

In papaya (*Carica papaya* L.) plants, the *DE-ETIOLATED-1* (*DET1*) gene, which is a negative regulator of photomorphogenesis, was suppressed through RNAi in embryonic callus to study its effects on the expression of a gene involved in the biosynthesis pathway of secondary metabolites, with results suggesting a relationship between the photo-regulated pathway and secondary metabolite synthesis [170].

## 3. Clustered Regularly Interspaced Short Palindromic Repeat (CRISPR)/CRISPR-Associated Protein (CRISPR/Cas)

**Figure 3:**
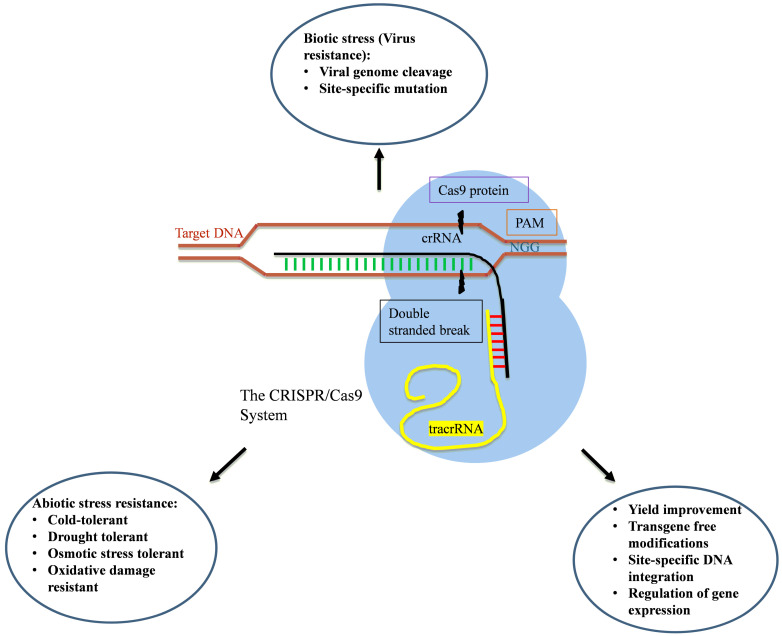
Applications of the CRISPR/Cas9 system in crop improvement. Here, the omission of undesirable traits and the adjunction of purposeful traits occurs through highly specific targeted genome modification.

Until 2013, the zinc-finger nucleases (ZFNs) and transcription activator-like effector nucleases (TALENs) were used as the most prevalent gene-editing tools [171,172,173]. These methods of gene editing rely on the use of specific DNA recognition and binding properties of specialized proteins viz. customized homing nuclease (meganuclease), zinc-finger nucleases (ZFNs), and transcription activator-like effector nucleases (TALENs). These nucleases are fusion products of domains derived from zinc finger transcription factors or transcription activator-like effectors formulated to identify almost any DNA sequence and the endonuclease domain of class II restriction enzyme, which can introduce double-stranded breaks (DSBs) [174]. CRISPR (Clustered Regularly Interspaced Short Palindromic Repeats) and the CRISPR-associated protein-9 (Cas9) nuclease system came out as a viable tool for targeted gene editing in plants [175]. In the last decade, the CRISPR/CAS system has made great achievements in many fields owing to its targeting, efficiency, versatility, and simplicity (Figure 3).

CRISPR comprises a DNA fragment with short palindromic repeats that are interspaced by the short sequences of variable length regarded as ‘non-repetitive’ elements or spacers. The CRISPR assembly was first recognized in the genome of *Escherichia coli* in 1987 [176]. The functional relationship between the CRISPR locus and adjacently located CRISPR-associated (Cas) genes was identified later [177]. The biological function of the CRISPR/Cas system was, however, unknown until 2015. A quantum leap came in the gene-editing technology with the findings that variable spacer sequences are derived from the foreign genome of virus and plasmid, suggesting the role of the CRISPR/Cas system as part of the adaptive immunity in prokaryotes [178,179,180]. The immunity is acquired by the acquisition of short DNA segments of invading viruses and plasmids in between the adjacent repeats as spacers. The CRISPR/Cas system provides immunity by utilizing the RNA-guided nucleases to cleave the genome of invaders in a sequence-specific manner. This was experimentally confirmed in 2007, i.e., phage-resistant bacteria have integrated spacers similar to the nucleic acid sequence of bacteriophage and the phage-resistant phenotype can be altered by the insertion or deletion of particular spacers. This implies that CRISPR, in association with Cas genes, could participate in providing immunity against viruses and plasmids [181]. The motifs associated with spacer precursor (proto-spacers) from the genome of invading viruses were identified at the time of the spacer uptake mechanism. These short stretches of di- or trinucleotides, which usually have sequence 5′-NGG- 3′ and exceptionally 5′-NAG-3′ present at one position downstream to proto-spacers, were named proto-spacers adjacent motifs (PAMs). These motifs (PAMs) play a key role in the identification of proto-spacers as well as assuring the correct integration of spacers in between repeated arrays of CRISPR [178,182,183]. Specificity is provided by the ‘Seed Sequence’ present approximately 12bp upstream of PAM, which must be complementary to the RNA. Brouns et al. [184] revealed that a long-transcript CRISPR RNA precursor (pre-crRNA) is produced by transcription of the CRISPR locus, which further processed into a mature crRNA molecule, which serves as single guide RNA (sgRNA). Each crRNA molecule consists of spacers, which are flanked by short DNA repeats, and this crRNA combines with transactivating CRISPR RNA (tracr RNA), which stimulate Cas9 and mediates the antiviral response. In 2010, it was experimentally proved that the CRISPR1/Cas system of *Streptococcus thermophiles* naturally uptakes spacers from a self-replicating plasmid containing antibiotic-resistant genes, provided to select transformed bacteria. They also examined in vivo that CRISPR1/Cas creates double-stranded breaks at specific sites within proto-spacers, suggesting the molecular basis of CRISPR/Cas system-mediated adaptive immunity [185]. As compared to ZFNs and TALENs, the construction of the CRISPR/Cas system is easier as it consists of just a Cas9 protein and a synthetic single-guide RNA (sgRNA), which needs to be designed complementarily to the target DNA sequence.

### 3.1. Mechanism of Action

Makarova et al. [186] classified the CRISPR/Cas system into three distinct polythetic classes, named Type I, II, and III. Cas1 and Cas2 serve as a vital constituent of all three systems as they play a crucial role in the integration of spacers in between the repeated array of CRISPR. Each system consists of its signature proteins and depends on these proteins to generate an immune response against the invading virus or plasmid [187]. In summary:

1. Type I systems contain signature protein Cas3 that consists of both helicase and DNase domains for the degradation of target [188]. Recently, six subtypes of the type I system (Subtype I-A to I-F) have been identified to contain a variable number of Cas proteins. Aside from Cas proteins, the type I system also encodes for the CRISPR-associated complex for the antiviral defense (Cascade) complex, and Cas3 is also the part of this complex.
2. Type II encodes three signature proteins, viz. Cas1, Cas2, and Cas9, and sometimes a fourth protein, i.e., Csn2 and Cas4. Cas9 is a multifunctional protein that plays a crucial role in the Type II system in adaptation to the degradation of the target along with trans-encoded small RNA (tracr RNA) [4,185,189,190]. Three subtypes of the type II system have been discovered, namely type II-A, type II-B, and type II-C [191,192].
3. Type III is defined by the presence of Cas10, whose function is still unclear. Two subtypes of the type III system (type III-A and type III-B) have been identified [193].

Type I and II systems target DNA degradation, but exceptionally, the type III system targets DNA as well as RNA. The most widely used system is the type II CRISPR/Cas9 system from *Streptococcus pyogenes* [4]. Until now, the type II system has been studied in bacteria but type I and type III systems have marked their presence in both bacteria and archaea [186]. The general mechanism of action of the CRISPR/Cas system involves three stages, i.e., adaption, expression, and interference. The proteins involved in the adaption stage (namely, Cas1 and Cas2) are highly conserved, whereas in expression and interference stages, the proteins vary greatly between the organisms. Each stage details are given below:

1. Adaption stage: The short pieces of DNA homologous to the genomic sequence of the invading virus or plasmid get incorporated at the leader side of the CRISPR locus. A new spacer unit is created by the duplication of repeats at every integration step. In type I and III CRISPR/Cas systems, the selection of proto-spacers occur by the recognition of PAMs present on or near the location of proto-spacers of the invading genetic element [183,194,195]. After the recognition, Cas1 and Cas2 proteins help in the integration of proto-spacers in between the repeat arrays of CRISPR.
2. Expression stage: At this stage, the expression of the spacer takes place via transcription of the CRISPR locus and leads to the generation of a long transcript of pre-CRISPR RNA (pre-crRNA), which is processed into short crRNA by endoribonucleases. In the type I CRISPR/Cas system, pre-crRNA binds with the CRISPR-associated complex for the antiviral defense (Cascade) complex, processed into crRNA by cleavage through Cas6e and Cas6f. The crRNA produced has an 8-nt repeat fragment at the 5′ end and the fragment left forms the hairpin structure on the 3′ end. In the type II CRISPR/Cas system, a repeated fragment of pre-crRNA pairs with the trans-encoded small RNA (tracer RNA), which is further cleaved by RNase III in the presence of Cas9 [189]. Consequently, cleavage at a fixed distance in spacers may lead to maturation. The type III system uses the Cas6 protein for processing to crRNA, but afterward, crRNA is transferred to a different complex of Cas proteins, namely Csm in subtype III-A systems and Cmr in Subtype III-B. Further, cleavage occurs at the 3′ end in subtype III-B subsystems [196].
3. Interference stage: After the expression, invading DNA or RNA is targeted and cleaved within proto-spacer sequences. The crRNA acts as a single guide RNA and guides the Cas protein towards the complementary target sequences of the invading genome of the virus or plasmid. In type I systems, the Cascade complex is guided by crRNA towards complementary target DNA, and invading DNA possibly cleaved by Cas3 protein. The Cas9 protein loaded with crRNA cleaves the target DNA in type II systems. The subtype of the type III system, III-A systems, target DNA [194] whereas III-B systems target RNA [196].

### 3.2. Applications

Progression of the CRISPR/Cas system from a biological defense phenomenon to a gene-editing tool came into light when it was revealed that the genome sequence can be remodeled by simply modifying the 20nt in the crRNA, and fusing it with tracr RNA to make a single chimeric guide RNA (gRNA). This led to the reduction of the three-component system to a two-component CRISPR/Cas system [4]. Unlike the Cas3 protein, which degrades the target completely, Cas9 introduces single double-stranded breaks (DSBs) in DNA, which is a salient feature to be an efficient gene-editing tool. DSBs induced in DNA triggers the DNA repair pathways in the cell, and CRISPR/Cas9 manipulates these pathways to alter the genome. Two main pathways are involved in the DNA repair, viz. non-homologous end joining (NHEJ) and homology-directed repair (HDR). NHEJ is more error-prone as while the cuts, insertion, or deletion (InDels) mutation may take place, gene knock-out of mutation occurs in the coding region or the production of a cripple gene product. HDR utilizes another piece of DNA homologous to target DNA to repair DSBs. As in HDR, the incorporation of a DNA element takes place through recombination, so any kind of insertion, deletion, or alteration in the sequence can be done [197,198]. Off-target cleavage can be avoided by selecting unique target sites adjacent to PAM [4]. These approaches have previously been studied using ZFNs and TALENs, but comparatively, Cas9 is simple to construct while it can also target multiple genes simultaneously [197].

Owing to its high efficiency, versatility, and simplicity, the CRISPR/Cas system can be employed for developing new cultivars (Table 2). It has been widely used in many plants such as *A. thaliana*, *N. benthamiana*, *O. sativa,* and *S. tuberosum* [199,200].

#### 3.2.1. Yield Improvement

In several plants, seasonal change in day-length may trigger flowering and day-length sensitivity, limiting their geographical range of cultivation. A CRISPR/Cas9-mediated mutation in *SPG5*, which is a repressor of florigen paralog and flowering, resulted in rapid flowering with enhancement in the compact determinate growth habit of field tomato [221]. Li et al. [222], using the RNA-guided Cas9 system, demonstrated that this technology can be used in vivo as the desired target mutator (DTM) to develop a mutated maize germplasm. For hybrid breeding in crops, photoperiod and thermosensitive genetic male sterility (PGMS and TGMS) are the two main components. To improve the yield potential of *O. sativa*, the development of hybrid *O. sativa* is important. Hybrid *O. sativa* breeding relies on the two-line system, and the generation of thermo-sensitive genetic male sterility is widely used in this system. TMS5 broadly applied the *TGMS* gene from China, which was manipulated using the CRISPR/Cas system to develop new ‘transgene clean’ TGMS lines. Eleven novel cultivars of TGMS were developed in one year, indicating the ability of the CRISPR/Cas system to improvieefficiency in hybrid *O. sativa* breeding [223]. Li et al. [224] employed a CRISPR/Cas gene-editing tool to mutate *Gna1*, *DEP1*, *GS3,* and *IPA1* genes of the *O. sativa* cultivar Zhonghua 11, resulted in T2 generation of *gna1*, *dep1,* and *gs3* mutants showing characteristics such as enhanced grain number, dense erect panicles, and larger grain size, respectively.

#### 3.2.2. Abiotic Stress Tolerance

The CRISPR/Cas9 system has enormous potential to generate crops tolerant to abiotic stresses. CRISPR/Cas9-mediated knock-out of the *O. sativa* annexin gene *OsAnn3* led to the development of mutant lines tolerant to cold, thus indicating the involvement of *OsAnn3* in cold tolerance of *O. sativa* [205]. In *A. thaliana*, C-repeat binding factors (CBFs) play a decisive role in cold-stress tolerance. However, the precise function of these factors is unclear owing to the lack of null cbf triplet mutants. Thus, CRISPR/Cas9 has been employed to produce cbf 1,3 double and cbf 1,2,3 triple mutants by disrupting *CBF1* or *CBF1*/*CBF2* in a *cbf3* T-DNA insertion mutant [225].

Mitogen-activated protein kinases (MAPK1) signaling molecules play a significant role in drought stress tolerance. In tomato, drought stress causes the accumulation of reactive oxygen species (ROS), which causes oxidative damage in tomatoes. *SlMAPK3* mutants generated by CRISPR/Cas gene editing led to more tolerance in the tomato plants [204]. Maize *ARGOS8* acts as a negative regulator of ethylene response. New variants of *ARGOS8* were developed with a native maize GOS2 promoter through CRISPR/Cas advanced breeding technology for the production of drought-tolerant crops. The field study showed that the *ARGOS8* variant of maize has increased grain yield significantly as compared to the wild type under flowering stress-condition [201].

SNF-1 related protein kinases 2 (SnRK2) serve as the main regulator of hyper-osmotic stress signaling and ABA-dependent development in plants. SnRK2 and osmotic stress/ABA activated protein kinase 2 (SAPK2) can be the primary mediator of ABA signaling in *O. sativa* subclass I and II. Lou et al. [226] examined the functional role of *SAPK2* by producing loss-of-function mutants using CRISPR/Cas technology. When drought, high-salinity, and polyethylene glycol (PEG) stresses were given, *SAPK2* expression was highly up-regulated. The *SAPK2* mutants showed an ABA-insensitive phenotype during germination and post-germination stages, suggesting the importance of ABA-mediated seed dormancy. Moreover, it has been observed that *SAPK2* increases the tolerance of *O. sativa* plants to salt and PEG stress.

#### 3.2.3. Biotic Stress Tolerance

The CRISPR/Cas9 system originally emerged as part of adaptive immunity in bacteria and archaea. Over the past years, it has been explored for targeted gene editing in various plants to provide resistance against biotic stresses. The CRISPR/Cas9 system was used to confer resistance against the *Tomato yellow leaf curl virus* (TYLCV) in *N. benthamiana* plants by designing sgRNA consisting of coding and non-coding sequences of TYLCV, resulting in reduced viral DNA accumulation with considerable attenuation in symptoms of infection [227]. Similarly, using the sgRNA-Cas9 system in *N. benthamiana beet severe curly top virus* (BSCTV) accumulation has also been reduced [228]. Virus-resistant cucumber (*Cucumis sativus* L.) cultivars were developed using the sgRNA-Cas9 system to disrupt the function of the recessive eIF4E gene. Resultant non-transgenic homozygous T3 progenies showed resistance against the *Cucumber vein yellowing virus* (Ipomovirus) infection, potyviruses such as the *Zucchini yellow mosaic virus* and the *Papaya ringspot mosaic virus-W* [203]. Likewise, Pyott et al. [202] employed CRISPR/Cas9 technology to introduce deleterious site-specific mutation in the *eIF(iso)4E* locus of *A. thaliana* to develop transgenic lines completely resistant against the *Turnip mosaic virus* (TuMV), which is a major pathogen for vegetables. These findings suggest that the CRISPR/Cas9 system is an innovative approach to generate potyvirus-resistant, agronomically important crops without incorporating transgenes. Zhan et al. [229] used CRISPR/Cas 13a in potato plants to develop resistance against the *Potato virus Y* (PVY). Reduced accumulation of virus and symptoms was observed in transgenic potato lines.

In *A. thaliana*, the *enhanced disease resistance 1* (*EDR1*) gene acts as a negative regulator of the defense response against powdery mildew. CRISPR/Cas9 technology was employed to develop the *Taedr1 Triticum* plants by altering the three homeologs of *Triticum EDR1*. No off-target mutations have been detected in *Taedr1* mutants and were found to be resistant to powdery mildew [230].

## 4. Conclusions and Future Prospects

**Figure 4:**
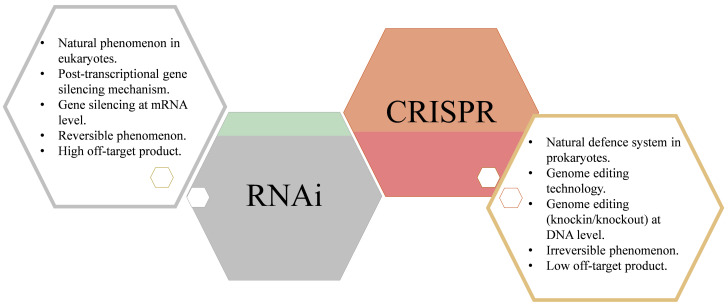
RNA interference vs. the CRISPR/Cas9 system.

In the 21st century, the foremost task for the agriculture industry is to provide food security to the rapidly expanding population globally. Besides, developing countries are also facing malnutrition. Hence, to ensure an adequate supply of balanced food to the world, there is an urgent need to develop biofortified staple food, vegetables, and fruits, enriched in all the essential compounds and mineral elements. The development of cultivars resistant to biotic stresses and tolerant to abiotic stresses i.e., changing environmental conditions, high temperature, drought, flood, oxidative stresses, high salt concentration, and heavy metal-polluted soil, can be a setback for world food security, malnutrition, and famine problems. The feasibility of using RNAi and CRISPR/Cas9 technology has become a topic of current interest in the last few years (Figure 4). These approaches hold great potential to develop crops with high-value agronomic traits by targeting their broad range of targets, accelerating crop improvement schemes, and increasing their effectiveness.

Current progress in CRISPR technology has advanced functional genomics research and innovative crop development. The recent technological advancement of CRISPR related to promoter, base editing, and prime editing has enhanced its effectiveness in gene editing for crop improvement [231]. The selection of the promoter for the expression of Cas9 and sgRNAs and the configuration of these expression cassettes are crucial to achieve proficient genome editing. Commonly, there are three expression strategies for the CRISPR-Cas system in plants, i.e., a mixed dual promoter system, a dual Pol II promoter system, and a single transcriptional unit system (STU) [232]. Although the STU system is considered superior over other two systems, it has limitations i.e., refinement of the system, nonoptimal expression system, and difficult post-transcriptional processes [232]. The limitations could be combatted by using a bidirectional promoter system that can initiate transcription in both orientations [233]. The two components (cas9 and sgRNA) are placed on each promoter’s end, enabling the opposite direction transcription. Further, the opposite architecture of the construct can help in balancing the expression strength and permit independent fine-tuning of the individual cas9 and sgRNA expression cassettes with the use of different enhancers, 3′ UTR, and terminators [234]. Furthermore, ‘base-editing’, i.e., the newest advancement of CRISPR-Cas-based technology, can be used to directly install point mutations in cellular DNA without inducing a double-strand DNA break [235]. The CRISPR/Cas-based, single-base-pair editing system can overcome limited efficiency and a high rate of undesired insertion or deletion mutations. The CRISPR/Cas base-editing system depends on the two classes of DNA base editors, i.e., adenine and cytosine base-editors, and is capable of conducting all four transition mutations (C→T, T→C, A→G, and G→A) [235]. At present, base-editing is considered as the most effective tool for plant genome editing. However, the base-editing technologies cannot generate precise base-edits beyond the four transition mutations. However, the recent technological evolution of prime editing of the CRISPER/Cas system can overcome the limitations [236]. Prime-editors consist of an engineered reverse transcriptase fused to Cas9 nickase and a prime-editing guide RNA. In prime-editing, a less stringent protospacer-adjacent motif is required due to the varied length of the reverse transcriptase template and no “bystander” editing [237]. However, prime-editing is still in its infancy, and there is a need to study its specificity and potential for off-target modification.

Moreover, both RNAi and CRISPR/Cas will bring a gene revolution in breeding crops with desired traits including quality. These technologies may help in supporting food security in both developed and developing countries. Studies concerned with gene silencing and gene deletion or disruption have also become essential to analyze the gene function in crops that could further help to design better gene-editing strategies.

## Figures and Tables

**Table 1:** Crops with improved stress tolerance through RNAi.

Trait(s)	Crop Improved	Resistance Against	Targeted Gene(s)	References
Virus resistance	*Nicotiana bethamiana*	Chilli-infecting begomoviruses	*AC1* *AC2* *βC1*	[43]
	*Triticum* spp.	*Triticum mosaic virus* (TMV)	*Coat protein* (*CP*)	[44]
	*Oryza sativa*	*Rice black streak dwarf virus* (RBSDV)	*S7-2* *S8*	[45]
	*Solanum tuberosum*	*Potato virus X* (PVX), *Potato virus Y* (PVY) *Potato virus S* (PVS)	*CP*	[46]
	*Glycine max*	*Soybean mosaic virus* (SMV)	*SMV P3* cistron	[47]
		*Mungbean yellow mosaic virus* (MYMIV)	*CP*	[48]
	*Arachis hypogaea*	*Tobacco streak virus* (TSV)	*CP*	[49]
	*O. sativa*	*Rice tungroo bacilliform virus* (RTBV) *Rice tungroo spherical virus* (RTSV)	*Coat protein 3 CP3*	[50]
	*Glycine max*	*Soybean mosaic virus* (SMV)	*eIF4E1*	[51]
	*N. bethamiana*	*Tomato yellow leaf curl Thailand virus* (TYLCTV)	*GSA*	[52]
Bacterial resistance	*A. thaliana*	*Agrobacterium tumefaciens*	*iaaM* *ipt*	[53]
		*Pseudomonas syringae*	*PPRL*	[54]
	*Citrus limon*	*Xanthomonas citri*	*CalS1*	[55]
Fungal resistance	*S. tuberosum*	*Phytophthora infestans*	*Avr3a*	[56]
	*T. aestivum*	*Fusarium graminearum*	*Chs 3b*	[57]
	*Musa* spp.	*F. oxysporum* f. sp. *cubense* (Foc)	*Foc velvet protein*	[58]
	*N. tabacum*	*Sclerotinia sclerotiorum*	*Chs*	[59]
	*S. lycopersicum*	*F. oxysporum*	*Fow2* *chs V*	[60]
	*O. sativa*	*Magnaporthe oryzae*	*MoABC1 MoMAC1 MoPMK1*	[61]
		*Rhizoctonia solani*	*RPMK1-1 RPMK1-2*	[62]
	*Zea mays*	*Aspergillus flavus*	*ZmPRms*	[63]
	*S. lycopersicum*	*F. oxysporum*	*Fmk1* *Hog1* *Pbs2*	[64]
	*Z. mays*	*A. flavus*	*Amy1*	[65]
	*S. tuberosum*	*Phytophthora infestans* *Alternaria solani*	*PVS1* *PVS2* *PVS3* *PVS4*	[66]
	*Glycine max*	*Phytophthora sojae*	*GmSnRK1.1*	[67]
	*S. lycopersicum*	*F. oxysporum*	*ODC*	[68]
Insect resistance	*S. lycopersicum*	*Helicoverpa armigera*	*HaCHI*	[69]
	*N. tabacum*	*Bemisia tabaci*	*AChE* *EcR*	[70]
	Lettuce	*B. tabaci*	*V-ATPase*	[71]
	*A. thaliana*	*Myzus persicae*	*MyCP*	[72]
	*Brassica rapa*	*Tetranychus urticae*	*COPB2*	[73]
Nematodes Resistance	*S. lycopersicum*	*Meloidogyne incognita*	*Mi-cpl1*	[74]
	*N. benthamiana*	*Radopholus similis*	*Rs-cps*	[75]
	*S. lycopersicum*	*M. incognita*	*PolA1*	[76]
	*Glycine max*	*Heterodera glycines*	*Hg16B09*	[77]
			*HgY25 HgPrp17*	[78]
	*A. thaliana*	*M. incognita*	*Mi-msp3* *Mi-msp 5* *Mi-msp18* *Mi-msp24*	[79]
Abiotic stress tolerance	*N. tabacum*	Salt tolerance	*Nt ε-LCY*	[80]
	*O. sativa*	Salt tolerance	*OsPEX11*	[81]
	*B. rapa*	Salt tolerance	*GIGANTEA* (*GI*)	[82]
	*A. thaliana*	Drought tolerance	*PAD4* *LSD1 EDS1*	[83]
	*O. sativa*	Drought tolerance	*OsGRXS17*	[84]
	*O. sativa*	Drought tolerance	*OsDSR-1*	[85]
	*O. sativa*	Drought tolerance	*OsERF101*	[86]
	*S. lycopersicum*	Drought and salt tolerance	*SlbZIP1*	[87]
	*N. tabacum*	Drought tolerance	*BrDST71*	[88]
	*T. aestivum*	Salt tolerance	*TaPUB-1*	[89]
	*A. thaliana*	Osmotic tolerance	*WZY2*	[90]

**Table 2:** CRISPR/Cas9 system-mediated gene editing in crops.

Trait(s)	Crop Used	Targeted Gene(s)	References
Drought tolerance	*Z. mays* (Maize)	*ARGOS8*	[201]
*Turnip mosaic virus* (TMV) resistance	*A. thaliana*	*eIF(iso)4E*	[202]
*Cucumber vein yellowing virus* (CMYV) resistance	*Cucumis sativus*	*eIF4E*	[203]
Drought tolerance	*S. lycopersicum*	*SlMAPK3*	[204]
Cold tolerance	*O. sativa*	*OsAnn3*	[205]
Parthenocarpic fruit development	*S. lycopersicum*	*SlIAA9*	[206]
Chilling stress tolerance	*S. lycopersicum*	*SlCBF1*	[207]
*Tomato yellow leaf curl virus* (TYLCV) resistance	*S. lycopersicum*, *N. benthamiana*	*Coat protein* (*CP*) *Replicase* (*Rep*)	[208]
*Cauliflower mosaic virus* (CMV) resistance	*A. thaliana*	*CaMV CP*	[209]
*Rice tungro spherical virus* (RTSV) resistance	*O. sativa*	*eIF4G*	[210]
Salt tolerance		*OsRR22*	[211]
Male-sterile development	*T. aestivum*	*Ms1*	[212]
Heat stress tolerance	*S. lycopersicum*	*SlMAPK3*	[127]
Drought and salt stress tolerance	*A. thaliana*	*DAP4* *SOD7*	[213]
Drought tolerance		*AREB1*	[214]
*Wheat dwarf virus* (WDV) resistance	*Hordeum vulgare*	*CP* *Rep*/*Rep4*	[215]
Yield improvement	*B. napus*	*BnaMAX1*	[216]
Yield improvement Stress tolerance	*O. sativa* (Nippobare)	*OsPIN5b* *GS3* *OsMYB30*	[217]
Yield improvement	*O. sativa*	*Cyt P450 homeologs* *OsBADH2*	[218]
Drought and stress tolerance		*OsDST*	[219]
*Tomato yellow leaf curl virus* (TYLCV) resistance	*S. lycopersicum*	*rgsCaM*	[220]
*Soyabean mosaic virus* (SMV) resistance	*Glycine max*	*GmF3H1* *GmF3H2* *GmFNSII-1*	[171]

## Data Availability

Not applicable.
